# Migrant Health and COVID-19 Pandemic: A Cross-sectional Study of Characteristics, Clinical Features, and Health Outcome from Iran

**DOI:** 10.1007/s44197-022-00063-3

**Published:** 2022-09-20

**Authors:** Mohammad-Reza Sohrabi, Rozhin Amin, Ali Maher, Shahriar Janbazi, Ali-Reza Zali

**Affiliations:** 1 Community Medicine Department, School of Medicine, Shahid Beheshti University of Medical Sciences, Tehran, Iran; 2 Social Determinants of Health Research Center, Shahid Beheshti University of Medical Sciences, Tehran, Iran; 3 School of Management and Medical Education, Shahid Beheshti University of Medical Sciences, Tehran, Iran; 4 Functional Neurosurgery Research Center, Shahid Beheshti University of Medical Sciences, Tehran, Iran

**Keywords:** COVID-19, Healthcare disparities, Health equity, Social determinants of health, Transients and migrants

## Abstract

**Background:**

As the pandemic unfolds, major concerns remain with those in disadvantaged positions who may be disproportionately affected. This paper aimed to present the characteristics of COVID-19 immigrant patients and investigate whether they were disproportionately affected by COVID-19 pandemic.

**Methods:**

A cross-sectional study was performed using data on 589,146 patients diagnosed with COVID-19 in Iran. Descriptive analyses were used to summarize the study population’s characteristics. Chi-squared test and logistic regression model were applied.

**Results:**

After accounting for possible confounding covariates, being an immigrant was significantly associated with increased risk of death due to COVID-19 (OR 1.64, CI 1.568–1.727). When compared to Iranian-born patients, the prevalence of low blood oxygen levels on admission was higher among immigrant patients (53.9% versus 47.7%, *P* value < 0.001). Moreover, greater proportions of immigrants who were diagnosed with COVID-19 were admitted to an ICU (17% versus 15.8%, *P* value < 0.001). Patients aged 65 and above were the largest age category in both populations. However, there was a significant difference between the age profiles of patients, with children under the age of eighteen presenting 16% of immigrant patients vs 6.6% of Iranian-born patients (*P* value < 0.001). In both groups, more men were affected by COVID-19 than women, yet the sex bias was more prominent for migrant patients (*P* value < 0.001).

**Conclusion:**

The evidence from this study revealed that immigrant patients infected with COVID-19 were more likely to suffer from severe health outcome of the disease compared to Iranian-born patients.

## Background

The ongoing pandemic of the new disease caused by Severe Acute Respiratory Syndrome Coronavirus 2 (SARS-CoV-2) has now affected the lives and mental health of all communities around the globe. The disease initially appeared in China in late 2019 and was named as coronavirus disease 2019 (COVID-19) by the World Health Organization (WHO) in early 2020 [1, 2]. By the time of writing, over 500 million confirmed cases of COVID-19 have been recorded worldwide including over six million deaths [3]. In Iran, the outbreak was first detected in a north-central province in February 2020. By early March, the virus had spread to all regions of the country, making it one of the first hard-hit countries by coronavirus in the world [4]. To date, the country’s total number of COVID-19 confirmed cases has surpassed seven million, and over 140000 confirmed deaths have been reported [5].

The COVID-19 pandemic has had unprecedented health and social consequences for almost all populations. Yet, as the pandemic unfolds, major concerns remain with those in disadvantaged positions who may be disproportionately affected. People who have been displaced, be it refugees, asylum seekers, migrants, or immigrants, represent one of the most important groups who are vulnerable to epidemics. Generally, these populations live in overcrowded conditions and poor-quality housings, making them prone to various communicable diseases. They often face challenges in accessing healthcare services due to multiple barriers including linguistic differences, financial instability, lack of legal status, and unawareness of their rights to healthcare [6]. In addition, chronic comorbid conditions which are not appropriately cared for, such as cardiac diseases, diabetes, and hypertension, may be more prevalent in them [7]. All these could pose a greater risk of morbidity and mortality on these populations at the time of pandemics.

Iran hosts one of the largest immigrants, in particular refugee populations worldwide [8]. The great majority coming from Afghanistan (about 97%) with the remaining being mostly Iraqi nationals. About one million registered Afghan nationals reside in Iran. However, estimates show that there are additional 2.1 million undocumented foreign nationals (mostly with Afghan nationality) who live in the country [9]. Registered immigrants are included in the national health system. Meaning that they have access to primary health care services at no charge and are eligible for enrollment in the national health insurance plans covering treatments and hospitalizations. However, in an inclusive response approach to COVID-19 epidemic in the country, all foreign nationals living in Iran, documented or undocumented, were provided with free of charge COVID-19 testing and hospital services [10]. Yet, there are still several barriers in receiving effective health care for people living with foreign nationalities in Iran. Barriers, such as cultural beliefs, low health literacy, and hesitancy to access care out of fears about immigration enforcement, which could make them more susceptible to the infection and its negative consequences [11].

Given the limited studies which have assessed the burden of the pandemic on displaced populations, this paper aimed to present the characteristics of COVID-19 immigrant patients residing in Iran and investigate whether they were disproportionately affected by COVID-19 pandemic.

## Methods

A population-based cross-sectional study was performed using data form the registry database of Coronavirus Control Operations Headquarter of Tehran, the most populated province of Iran. With over thirteen million residences, the province is an important epicenter for COVID-19 epidemic in the country [12]. The integrated COVID-19 registry was established in the province in March 2020, and data on all patients diagnosed with COVID-19 were documented ever since based on WHO case definition guidance [13]. In this descriptive multi-center study, all patients with SARS-CoV-2 infection (589,146 individuals) who were seen in healthcare facilities across the province of Tehran from March 2020 up to November 2021 were included. Of those, 256,359 (43%) patients had positive PCR test results for SARS-CoV-2, and for the remaining, the diagnosis was established based on chest CT findings and clinically epidemiologically criteria. Overall, data on demographic characteristics, clinical manifestations, underlying diseases, para-clinical findings, and the health outcome of 589,146 patients were extracted and anonymized from the records.

### Variables

Data included information on age, sex, smoking history, opioids history, underlying diseases, clinical and para-clinical findings, intensive care unit (ICU) admission, and the disease outcome. Underlying diseases included diabetes, hypertension, cardiovascular diseases (CVD), cancer, asthma, chronic liver diseases, chronic kidney diseases, chronic neurological diseases, chronic hematological diseases, and chronic immune deficiency diseases. Clinical manifestations included fever, cough, muscle ache, difficulty breathing, chest pain, loss of smell, loss of taste, loss of appetite, nausea, diarrhea, headache, vertigo, seizure, paraplegia, and skin lesions. Blood oxygen saturation level, polymerase chain reaction (PCR) test result, and chest CT scan report were described as para-clinical findings.

Age was categorized into 9 groups: 0–4, 5–11, 12–17, 18–24, 25–34, 35–44, 45–54, 55–64, and 65 years or more. Sex was classified as female or male. The blood oxygen saturation level was defined as being either higher than 93% or 93% and lower, according to the National Coronavirus Control Operations Headquarter protocol. PCR test result was defined as negative, inconclusive, and positive. The chest CT scan report was divided into reports with positive COVID-19-related findings and those with no COVID-19 findings. The disease outcome was described as survived or deceased. All other variables were dichotomized as yes or no.

### Statistical Analysis

Descriptive analyses were used to summarize the study population’s characteristics. Chi-squared test was performed to assess the differences in patients’ characteristics, clinical and para-clinical findings, and the health outcome between the immigrant and Iranian-born patients. The logistic regression model was applied to adjust for possible confounding effect of variables including demographic and existing comorbidities on the health outcome of patients. Estimates were examined with *P* value < 0.05 indicating a significant difference and using IBM SPSS Statistics, version 27 (IBM Corp., Armonk, N.Y., USA). Since the information was documented by trained healthcare personnel, the rates of missing values were low across all variables used in this study. Therefore, the impact of missing information on the statistical inferences drawn from the data was considered as insignificant [14].

## Results

In this study, a total of 589,146 patients (20,992 immigrant patients, 568,154 Iranian patients) who were diagnosed with COVID-19 were included. The mean ± SD age was 42 ± 23.4 years for immigrant patients and 51 ± 20.9 for Iranian patients. Patients aged 65 and above were the largest age category in both populations. However, there was a significant difference between the age profile of patients, with children under the age of eighteen presenting 16% of immigrant patients vs 6.6% of Iranian patients. In both groups, more men were affected by COVID-19 than women, yet the sex bias was more prominent for immigrant patients. The prevalence of smoking was almost the same in both populations, while the proportion of patients using opioids was higher for immigrant patients. Comorbidities including diabetes, hypertension, CVD, cancers, and chronic neurological disorders were less prevalent in patients with foreign nationality residing in the country. However, no significant difference was observed for the prevalence of asthma, chronic kidney diseases, chronic liver diseases, chronic immune deficiency disorders, and chronic hematological diseases between the two groups of patients. Significant differences were noted in the distribution of the clinical manifestations between the two groups, except for difficulty breathing and paraplegia (Table 1).

**Table 1:** Characteristics and clinical presentations of patients infected with COVID-19, Iran, 2020–2021

Characteristics	Iranian patients		Immigrant patients		*P* value
	*n*	%	*n*	%	
*Age (years)*					
0–4	25,262	4.4	2200	10.5	< 0.001
5–11	7408	1.3	717	3.4	
12–17	4918	0.9	448	2.1	
18–24	13,442	2.4	1359	6.5	
25–34	56,466	9.9	2758	13.1	
35–44	94,617	16.7	3106	14.8	
45–54	95,896	16.9	3198	15.2	
55–64	105,851	18.6	3139	15.0	
65 and over	164,294	28.9	4067	19.4	
*Sex*					
Female	270,578	47.6	9636	45.9	< 0.001
Male	297,576	52.4	11,356	54.1	
Positive history of smoking	8618	1.5	290	1.4	0.11
Positive history of opioids	4434	0.8	268	1.3	< 0.001
*Underlying diseases*					
Diabetes	56,119	9.9	1224	5.8	< 0.001
Hypertension	64,053	11.3	1431	6.8	< 0.001
CVD	52,465	9.2	1031	4.9	< 0.001
Cancer	8891	1.6	225	1.1	< 0.001
Asthma	5927	1.0	215	1.0	0.79
Chronic liver diseases	2435	0.4	105	0.5	0.12
Chronic kidney diseases	9012	1.6	293	1.4	0.03
Chronic neurological diseases	4059	0.7	112	0.5	0.002
Chronic immune deficiency diseases	1547	0.3	43	0.2	0.06
Chronic hematological diseases	2562	0.5	95	0.5	0.97
*Clinical presentations*					
Fever	210,294	37.0	7267	34.6	< 0.001
Cough	295,532	52.0	9020	43.0	< 0.001
Muscle ache	191,694	33.7	5608	26.7	< 0.001
Difficulty breathing	240,796	42.4	8828	42.1	0.34
Chest pain	16,363	3.1	507	2.6	< 0.001
Loss of smell	14,365	2.5	257	1.2	< 0.001
Loss of taste	8393	1.5	182	0.9	< 0.001
Loss of appetite	45,292	8.4	1216	6.2	< 0.001
Nausea	36,046	6.7	1090	5.6	< 0.001
Diarrhea	19,052	3.5	785	4.0	< 0.001
Headache	48,991	9.1	1032	5.4	< 0.001
Vertigo	15,810	2.9	420	2.2	< 0.001
Seizure	1920	0.3	241	0.1	< 0.001
Paraplegia	717	0.1	21	0.1	0.36
Skin lesions	635	0.1	41	0.2	< 0.001

More than half of the immigrant patients had low levels of blood oxygen saturation on admission. The rate was significantly lower in patients with Iranian nationality. Of the immigrant patients, about a third had a positive SARS-CoV-2 PCR testing result, whereas almost half of the Iranian patients had positive PCR test results for SARS-CoV-2. More Iranian patients had chest CT reports indicating COVID-19-related findings compared to immigrant patients. However, higher rates of ICU admission and death due to COVID-19 were documented for foreign nationals when compared to Iranians (Table 2).

**Table 2:** Para-clinical findings and health outcome of patients infected with COVID-19, Iran, 2020–2021

	Iranian patients		Immigrant patients		*P* value
	*n*	%	*n*	%	
PaO_2_ sat < 93%	270,811	47.7	11,314	53.9	< 0.001
Positive PCR test result	250,649	44.1	5710	27.2	< 0.001
Chest CT with positive findings	375,468	66.1	11,369	54.2	< 0.001
ICU admitted	90,044	15.8	3576	17.0	< 0.001
Deceased	48,765	8.6	2127	10.1	< 0.001

After accounting for possible confounding covariates including age, sex, history of smoking, history of opioids, and underlying diseases in the logistic regression model, the observed disparity in proportions of deceased patients remained significant. Being an immigrant was significantly associated with increased risk of death with COVID-19 (OR 1.64, CI 1.568–1.727) when compared to Iranian patients (Table 3).

**Table 3:** Logistic regression model of independent variables associated with COVID-19-related death, Iran, 2020–2021

variable	aOR	95% confidence interval		*P* value
		Lower	Upper	
*Age group*				
0–4	1			
5–11	0.38	0.316	0.469	< 0.001
12–17	0.71	0.595	0.858	< 0.001
18–24	0.53	0.466	0.612	< 0.001
25–34	0.50	0.461	0.552	< 0.001
35–44	0.84	0.786	0.914	< 0.001
45–54	1.49	1.387	1.600	< 0.001
55–64	2.68	2.509	2.879	< 0.001
65+	6.20	5.799	6.630	< 0.001
*Sex*				
Female	1			
Male	1.31	1.288	1.338	< 0.001
*Nationality*				
Iranian patients	1			
Immigrant patients	1.64	1.568	1.727	< 0.001
Positive history of smoking	0.80	0.740	0.865	< 0.001
Positive history of opioids	1.31	1.205	1.443	< 0.001
Diabetes	1.16	1.129	1.195	< 0.001
Hypertension	1.04	1.012	1.068	0.004
CVD	1.15	1.126	1.190	< 0.001
Cancer	2.21	2.097	2.339	< 0.001
Asthma	0.91	0.842	1.001	0.05
Chronic liver diseases	1.63	1.458	1.824	< 0.001
Chronic kidney diseases	2.02	1.916	2.129	< 0.001
Chronic neurological diseases	1.78	1.646	1.941	< 0.001
Chronic immune deficiency diseases	1.28	1.084	1.527	0.004
Chronic hematological diseases	1.40	1.248	1.579	< 0.001
Constant	0.28			

## Discussion

The evidence from this study revealed that immigrant patients infected with COVID-19 were more likely to suffer from severe health outcomes of the disease compared to Iranian-born patients. The main findings indicated that being an immigrant was significantly associated with increased risk of dying with COVID-19. More immigrant patients presented with low blood oxygen levels on admission and required ICU care when compared to Iranians.

Overall, there were pronounced differences in health outcome between the two groups, with migrant patients having higher rates of ICU admission and death due to COVID-19. After accounting for possible confounding factors, the probability of COVID-19-related mortality was also higher among immigrants compared to Iranian patients. The disproportionate burden of the disease found in this study confirms the results reported from other countries [15–18]. The poorer health outcome of people with migrant status is frequently attributed to multiple barriers which hinder their access to health care, and result in accessing care when the disease is more advanced compared to the host population. Barriers are, such as differences in language and cultural beliefs, lack of entitlement to health care, and reluctance to use health services out of fears about one’s legal status [19–21]. However, immigrants in Iran are mostly from the neighboring country; Afghanistan; and share the same language and cultural values with Iranian-born population. Moreover, as of March 2020, all foreign nationals irrespective of their legal status, were entitled to free of charge COVID-19 testing and hospital services in an inclusive approach to the epidemic response [22]. Hence, the disparities in the health outcome between immigrant and Iranian patients which were observed in this study, might point to more distant determinants of health which could affect the course of the disease and ultimately influence the outcome of patients infected with COVID-19. Immigrants in Iran could generally be characterized as low-income communities with poor overall health status [23]. Many send most of their earnings to their home country to support their families, which leaves them unable to afford non-COVID-19 health services, therefore, they often suffer from chronic conditions which are either not diagnosed, or not properly managed. Moreover, most labor migrants are day workers who would lose the day’s pay if left work to get tested or see physicians. All these could result in delayed diagnosis of SARS-CoV-2 infection, exacerbate the severity of the disease, and increase the risk of morbidity and mortality in the migrant population.

Overall, patients with non-Iranian nationality living in the country were younger than Iranian patients, which was in line with findings from immigrant populations who were infected with COVID-19 in Kuwait, Italy, and United States [15–17]. This result is not particularly surprising given the fact that most foreign nationals in Iran are migrant workers. With respect to gender composition, patients were less frequently women in both Iranian and non-Iranian populations which was consistent with reports around the world [15, 17, 18, 24, 25]. However, the difference was more prominent for immigrant patients, which could be partly explained by the great proportion of labor migrants (mainly men) among non-Iranian patients. However, biological variations between men and women which could make men more susceptible to SARS-CoV-2 infection, as well as differences in behavioral habits between the two sexes have also been mentioned in the literature to justify the sex bias observed in COVID-19 pandemic [26, 27].

The most prevalent underlying diseases were hypertension and diabetes in both groups which correlate well with those of other countries and further support the potential role of angiotensin-converting enzyme 2 (ACE2) receptors in the coronavirus entry into human cells [28–31]. Yet the conditions were more prevalent in the patients with Iranian nationality. Higher prevalence of hypertension, diabetes, and several other comorbidities in Iranian patients compared to immigrant patients living in the country, could be attributed to the relatively older age of patients with Iranian nationality. However, another possible explanation for the observed discrepancy could be the underdiagnosis of chronic conditions in immigrant population.

In agreement with findings documented in previous studies, in both groups, respiratory symptoms including cough and difficulty breathing were the most common complaints that have prompted patients to seek medical care [32, 33]. However, it is not completely clear why most symptoms are more prevalent in Iranian-born patients compared to immigrant population.

However, our research might have limitations. First, data on determinants, such as reason for relocation, migration status, and length of stay in Iran, which could influence the health outcome of immigrant patients infected with COVID-19 in the country, were not available to the researchers. Second, since we did not have information on socio-economic status of the patients, we have compared immigrant patients of whom mostly are from low socio-economic status with the general population of Iran that has socio-economic diversity. If we assume that socio-economic status could impact the health outcome of patients with COVID-19, then comparing immigrant patients with Iranian patients from low socio-economic status might attenuate the observed disparity in the health outcome. As more comprehensive data become available, further research is needed to test this hypothesis. Yet, using real-time and consistent data on a large multi-center cohort of COVID-19 patients is the major strength of our study. This allows reliable extension of research findings to the target population and makes this research of great importance for policy-makers.

## Conclusion

The evidence from this study revealed that immigrant patients infected with COVID-19 were more likely to suffer from severe health outcomes of the disease compared to Iranian-born patients. The observed health disparity could be explained by several socio-economic health determinants which require targeted public health interventions aimed at reducing the health inequalities. All in all, immigrants are among vulnerable populations who demand special consideration from policy-makers in their response to COVID-19 pandemic. In addition to inclusive policies which ensure their entitlement to COVID-19 healthcare, and their inclusion in the national surveillance systems, it is particularly important to address the socio-economic health determinants which affect the overall health status in this population with social services that are tailored to their needs.

## Data Availability

The data that support the findings of this study are available from Coronavirus Control Operations Headquarter in the province of Tehran, but restrictions apply to the availability of these data, which were used under license for the current study, and so are not publicly available. Data are however available from the authors upon reasonable request and with permission of Coronavirus Control Operations Headquarter in the province of Tehran.

## Abbreviations

SARS-CoV-2: Severe acute respiratory syndrome coronavirus 2
COVID-19: Coronavirus disease 2019
WHO: World Health Organization
ICU: Intensive Care Unit
CVD: Cardiovascular diseases
PCR: Polymerase chain reaction
ACE2: Angiotensin-converting enzyme 2
